# The impact of pre-transplant donor specific antibodies on the outcome of kidney transplantation – Data from the Swiss transplant cohort study

**DOI:** 10.3389/fimmu.2022.1005790

**Published:** 2022-09-21

**Authors:** Lukas Frischknecht, Yun Deng, Caroline Wehmeier, Olivier de Rougemont, Jean Villard, Sylvie Ferrari-Lacraz, Déla Golshayan, Monique Gannagé, Isabelle Binet, Urs Wirthmueller, Daniel Sidler, Thomas Schachtner, Stefan Schaub, Jakob Nilsson, the Swiss Transplant Cohort Study, Patrizia Amico

**Affiliations:** ^1^ Department of Immunology, University Hospital Zurich (USZ), Zurich, Switzerland; ^2^ Clinic for Transplantation Immunology and Nephrology, University Hospital Basel, Basel, Switzerland; ^3^ Department of Surgery and Transplantation, University Hospital Zurich, Zurich, Switzerland; ^4^ Transplantation Immunology Unit and National Reference Laboratory for Histocompatibility, Department of Diagnostic, Geneva University Hospitals, Geneva, Switzerland; ^5^ Transplantation Center, Lausanne University Hospital, Lausanne, Switzerland; ^6^ Service of Immunology and Allergy, Lausanne University Hospital, University of Lausanne, Lausanne, Switzerland; ^7^ Nephrology & Transplantation Medicine, Cantonal Hospital St. Gallen, St. Gallen, Switzerland; ^8^ Department of Laboratory Medicine, Inselspital, Bern University Hospital and University of Bern, Bern, Switzerland; ^9^ Department of Nephrology and Hypertension, Inselspital, Berne University Hospital and University of Berne, Berne, Switzerland; ^10^ Division of Nephrology, University Hospital Zurich, Zurich, Switzerland

**Keywords:** kidney transplantation, donor specific antibodies, abmr, graft loss, virtual cross-match

## Abstract

**Background:**

Pre-transplant donor specific antibodies (DSA), directed at non-self human leukocyte antigen (HLA) protein variants present in the donor organ, have been associated with worse outcomes in kidney transplantation. The impact of the mean fluorescence intensity (MFI) and the target HLA antigen of the detected DSA has, however, not been conclusively studied in a large cohort with a complete virtual cross-match (vXM).

**Methods:**

We investigated the effect of pre-transplant DSA on the risk of antibody-mediated rejection (ABMR), graft loss, and the rate of eGFR decline in 411 DSA positive transplants and 1804 DSA negative controls.

**Results:**

Pre-transplant DSA were associated with a significantly increased risk of ABMR, graft loss, and accelerated eGFR decline. DSA directed at Class I and Class II HLA antigens were strongly associated with increased risk of ABMR, but only DSA directed at Class II associated with graft loss. DSA MFI markedly affected outcome, and Class II DSA were associated with ABMR already at 500-1000 MFI, whereas Class I DSA did not affect outcome at similar low MFI values. Furthermore, isolated DSA against HLA-DP carried comparable risks for ABMR, accelerated eGFR decline, and graft loss as DSA against HLA-DR.

**Conclusion:**

Our results have important implications for the construction and optimization of vXM algorithms used within organ allocation systems. Our data suggest that both the HLA antigen target of the detected DSA as well as the cumulative MFI should be considered and that different MFI cut-offs could be considered for Class I and Class II directed DSA.

## Introduction

Kidney transplantation is currently the preferred treatment option for end stage kidney disease, with over 100 000 transplantations performed globally each year. Significant improvements in pre- and post-transplant management during the last decades have led to impressive graft survival rates during the first years after transplantation (1). Long-term graft outcomes have, however, not markedly improved, and graft rejection resulting from antibody-mediated immune responses, directed at non-self human leukocyte antigen (HLA) proteins present in the graft, continue to be the main cause of graft loss at later time points (2–4). Antibody mediated rejection (ABMR), once established, is also difficult to treat, and despite intensified efforts, there are currently no available treatment options that have shown an ability to impact transplant outcome in a significant way (5–8). This highlights the importance of preventing the occurrence of ABMR by identifying patient and donor constellations at increased risk. The most important pre-transplant risk factor for the development of AMBR is the presence of donor specific antibodies (DSA) that target the non-self HLA protein variants in the intended donor (9). Current techniques utilizing single antigen bead (SAB) assays, where a single HLA protein variant is immobilized on a solid bead, are able to detect such antibodies with a high sensitivity (10). Anti-HLA antibodies can, however, be detected in a large portion of patients in need of a kidney transplant which severely limits transplant opportunities. By employing pre-transplant SAB assays at regular individualized intervals, in combination with modern qPCR based donor intermediate resolution HLA typing, a virtual cross-match (vXM) can be performed, and transplantations with DSA can be avoided (11, 12). In order to improve vXM strategies it is of crucial importance to accurately quantify the risk associated with pre-transplant DSA, so that this can be balanced against other clinically relevant risks. Several previous studies have clearly shown an increased risk of ABMR and graft loss in DSA positive transplantations both in the setting of pre-transplant DSA as well as *de novo* DSA (9, 13, 14). The long-term impact on transplant outcome of DSA directed at all individual HLA loci as well as the effect of the mean fluorescent intensity (MFI) of such DSA has, however, not been extensively studied in a large cohort of kidney transplant patients with a complete vXM and detailed data on long-term transplant outcome for up to 12 years. In order to improve pre-transplant immunological risk stratification, we investigated the effect of pre-transplant DSA in 411 DSA positive transplants within the Swiss Transplant Cohort Study (STCS).

## Materials and methods

### Study design and patient population

The study (project number FUP142) was nested within the Swiss Transplant Cohort Study (STCS), which is a prospective nationwide longitudinal cohort study in solid organ and stem cell transplantation in Switzerland. For our sub-study, we included patients receiving a kidney transplant between May 2008 and December 2017 in Switzerland (15). The Cantonal Ethics Committee of Zurich (BASEC-Nr.2021-0083) separately approved this sub-study.

**Figure 1:**
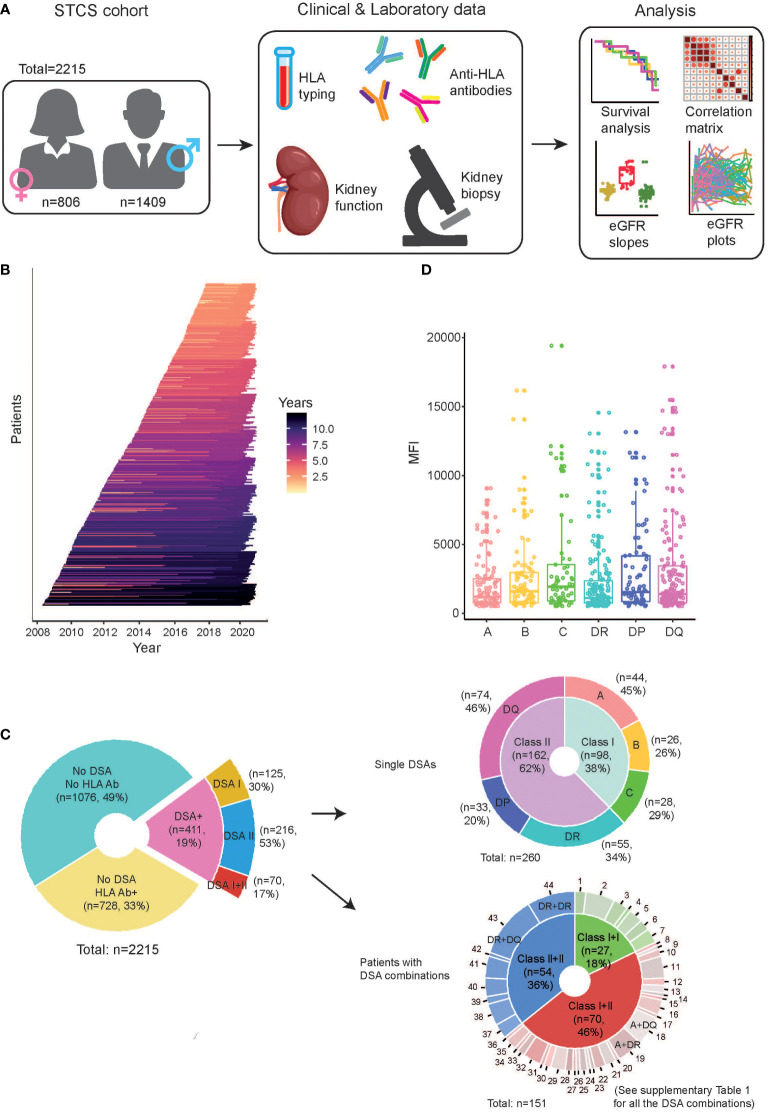
Characteristics of the study cohort. **(A)** Schematic of the workflow illustrating the study overview, the data collection from the clinical and laboratory observations, and main types of analyses in this study. **(B)** The timeline of the study follow-up on individual patients included after kidney transplantation. The individual lines are colored according to length of follow-up. **(C)** Overview of the patients with regard to the presence of HLA antibodies (HLA Ab) and DSA. In the samples containing DSA, they are further stratified as “single DSAs”, which refer to patients having only one DSA, as well as “patients with DSA combinations”, which refer to patients having multiple DSA. **(D)** The distribution of MFI of each DSA directed against individual HLA loci in the investigated patients.

A total of 2874 kidney transplantations were performed in Switzerland during the inclusion period, and 2657 were included in the STCS. For our sub-study, 442 transplants were excluded from the data analysis, which gives a total number of 2215 transplants (n=2179 patients) for the final analysis. Patients excluded from the study included those with the following conditions; 1) no baseline data prior to the transplantation (n=10), 2) having multi-organ transplants (n=158), 3) pediatric recipients (age <18yr) (n=90), 4) transplants with incomplete virtual crossmatch (n=28), 5) loss of follow-up before the end of first year (n=3), 6) ABO-incompatible transplants (n=153) (**Figures 1A, B**). The follow-up data on patients post-transplantation was collected at month 6 post-transplant and then continuously on an annual basis. Primary outcomes were ABMR, death censored graft survival and decline in graft function.

### Detection of HLA antibodies and DSA assignment

HLA antigens were determined by DNA based HLA-typing using either sequence-specific oligonucleotide (SSO) or sequence-specific primer (SSP) technologies. Apart from the standard HLA typing of donors, additional typing was performed if needed for any additional loci if the recipient had anti-HLA antibodies targeting an HLA locus that was not previously typed. This resulted in a complete vXM pre-transplant for all patients included in the final analysis.

In the majority of the transplants (99.6%), the presence of HLA antibodies was detected using a Luminex bead-based platform, while a few were detected by ELISA (0.4%). In this study, the majority of the included patients were analyzed with single-antigen bead (SAB) analysis directly before transplantation (LABScreen Single Antigen; OneLambda) while for the rest, a screening with mixed bead analysis (LABScreen Mixed, OneLambda) was first performed, and then subsequent SAB testing was done on all positive screens. In total 27% of the included transplants had a negative LABScreen Mix analysis and were considered anti-HLA antibody negative based on the Mix result. The fluorescence of the individual bead was detected by a reader (LABScan) and recorded as mean fluorescence intensity (MFI). Both historical and current HLA antibodies with a mean fluorescence intensity (MFI) > 500–1000 (depending on the center-specific cutoff) were included, with the majority of centers reporting antibodies >500 MFI.

As detailed in **Figure 1C**, 411 out of 2215 transplants were considered to have HLA-DSA as determined by virtual cross-matching using a direct comparison of the donor’s HLA typing with the recipient’s HLA antibody specificities. Cumulative DSA MFI was calculated by adding the MFIs of all detected DSA at HLA antigen resolution. The highest single DSA MFI was calculated by taking the MFI of the highest detected DSA at HLA antigen resolution.

### Diagnosis of rejection and definition of graft loss

All patients with documented rejection episodes were biopsy-proven. Biopsy specimens were obtained and evaluated, according to the local protocol, by treating physicians at the six Swiss transplant centers. Findings were recorded either by the individual Banff scores or as text, which was later translated and graded according to the 2017 Banff criteria (16). Biopsies with findings of “borderline changes” and “C4d positive staining without evidence of rejection” were not considered as rejection in our study (15). Graft loss was defined as return to dialysis or preemptive re-transplantation before dialysis was needed. Only death censored graft loss was used as an outcome parameter in our study.

### Calculation of eGFR and eGFR slope

Estimated glomerular filtration rate (eGFR) was calculated using the Chronic Kidney Disease Epidemiology Collaboration 2009 (CKD-EPI) creatinine equation (17). In total, 69 patients had records containing eGFR values that were considered outside of the normal diagnostic range, and these patients were therefore excluded from the eGFR analyses. The slope of the eGFR decline was determined using the eGFR value at 1 year post-transplant as baseline value. The individual slope was divided by the individual eGFR at baseline and defined in units of “ml/min/1.73m^2^/year”. In the longitudinal eGFR slope, the mean annual slope was calculated for each year of follow-up in the respective groups of patients. The mean total slope was calculated by using the last recorded eGFR value in each individual patient.

### Data processing and statistical analysis

Raw data were collected in Microsoft Office Excel. It was pre-processed with R (version 4.0.3) and RStudio (version 1.3.1093) using packages “dplyr” (1.0.7), “lubridate” (1.8.0), ”pacman” (0.5.1), “rio” (0.5.29), “tibble” (3.1.6) and “tidyr” (1.1.4). Missing values were omitted. Statistical significance was calculated with a (1) log-rank test to compare the Kaplan-Meier survival analysis between groups, (2) Mann-Whitney U test to analyze unpaired data with a non-Gaussian distribution for 2 groups’ comparison, (3) One-way analysis of variance (ANOVA) followed by Dunn’s *post hoc* test, or (4) Two-way ANOVA with Sidak’s (or Tukey’s) multiple comparisons as *post hoc* test for multiple comparisons in the plots for eGFR slope analysis, if not otherwise specified. The black circles in the violin plots indicate mean values in the respective group. All data points in the box plots are displayed with median and interquartile range, indicated with horizontal lines. Correlation analyses were done between various variables and quantified with Spearman’s correlation test. Statistical analyses and figure illustration were performed using R packages, including “corrplot” (0.92), “ggplot2” (3.3.5), “stats” (4.0.3) and “survival” (3.2.7). For univariate analyses, a Cox proportional hazards regression model was used. Hazard ratios, the corresponding 95% confidence interval (CI) and p values were estimated for each individual variable in relation to graft loss. To perform the multivariate analysis and overcome the multi-collinearity between the variables, a partial least squares (PLS) regression was used to model the dependence relationship between one dependent outcome variable and multiple independent variables in an exploratory fashion.

## Results

### Study population characteristics

The study population consisted of 2215 kidney transplantations performed in Switzerland between 2008 and 2017. An overview of study parameters is shown in **Table 1** and **Figure 1A**. The median follow-up time of the study population was 6.1 years, and 62% (n=1372) of the included patients underwent a kidney biopsy during the follow-up period (**Figure 1B**). Anti-HLA antibodies were detected in 1139 patients (51%) prior to transplantation (**Figure 1C**). In total, 411 of 2215 transplants (19%) were performed with a pre-transplant DSA. The DSA were most commonly directed at HLA Class II, and the majority of the detected DSA had an MFI below 2000 (**Figures 1C, D**). As expected, patients in the DSA positive group were more often female and had more frequently received a previous transplant (**Table 1**). The majority of the DSA positive patients had a single DSA (260/411, 63%), whereas the rest had multiple DSA with many directed at two or more HLA loci (**Figure 1C**). The most common combinations in the group with DSA against multiple loci were DR, DQ (12%), DR, DR (9%), and A, DQ (5%) (**Figure 1C**; **Supplementary Table 1**). A complete compilation of all occurring DSA combinations is present in **Supplementary Table 1**. The mean MFI of the detected DSA did not significantly differ between the different HLA target loci, but there was a slight trend towards higher DSA against HLA-DQ (**Figure 1D**).

**Table 1:** Characteristics of the recipient and donor in transplants with and without pre-transplant DSA.

Characteristics	DSA (n = 411)	No DSA (n = 1804)	p value (DSA vs. No DSA)
Female gender (Recipient)	197 (48%)	609 (34%)	<0.0001
Age at transplantation (mean value)	51.7	52.8	0.04
Female gender (Donor)	190 (46%)	934 (52%)	<0.0001
Age (Donor) (mean vlaue)	51.5	52.5	
Previously transplanted	181 (44%)	221 (12%)	<0.0001
Previous pregnancy	45 (23% in female)	130 (21% in female)	
Previous blood transfusion	204 (50%); 62 (unknown)	479 (27%); 341 (unknown)	
**Immunosuppression**			<0.0001
FK-MPA-Pred	354 (86%)	1364 (77%)	
CyA-MPA-Pred	44 (11%)	357 (20%)	
CNI-based other	10 (3%)	19 (1%)	
mTOR-containing	2 (1%)	48 (3%)	
Other	1 (0%)	14 (0%)	
**Induction therapy**			<0.0001
ATG/Thymo+/- lvlg	275 (67%)	274 (16%)	
Basiliximab	135 (33%)	1473 (81%)	
None	1 (0%)	57 (3%)	
**Underlying renal disease**			<0.0001
Glomerulonephritis	92 (22%)	449 (25%)	
ADPKD	75 (18%)	341 (19%)	
Diabetic nephropathy	28 (7%)	161 (9%)	
Vascular nephropathy	30 (7%)	222 (12%)	
Interstitial nephropathy	10 (3%)	63 (4%)	
Other	100 (24%)	403 (22%)	
Not specified	58 (14%)	211 (12%)	
Reflux/Pyelonephritis	18 (4%)	93 (5%)	
Hereditary (not ADPKD)	11 (3%)	57 (3%)	
Congenital	13 (3%)	42 (2%)	
Unknown	76 (19%)	165 (9%)	
**HLA mismatch**			
A, % with 0/1/2	14/47/39	16/46/38	0.800
B, % with 0/1/2	6/41/53	9/40/51	0.180
DRB1, % with 0/1/2	10/56/34	18/53/29	0.002
Cold ischemia time (DD)/h (mean value)	9.0 (6.9–12.1)	9.4 (7.3–12.3)	0.10
**Donor type**			
No. of donation (DD/LD)	294/117	1118/686	<0.0001
Percentage. of donation (DD/LD), %	71.5/28.5	62.0/38.0	<0.0001
Age donor (DD/LD) (mean value)	50.7/53.5	51.9/53.6	0.362
Female gender of donor (DD/LD), %	42.5/55.6	44.2/64.4	0.098

DD, Disease donation; LD, Living donation.

### The presence of pre-transplant DSA negatively affects transplant outcome

**Figure 2:**
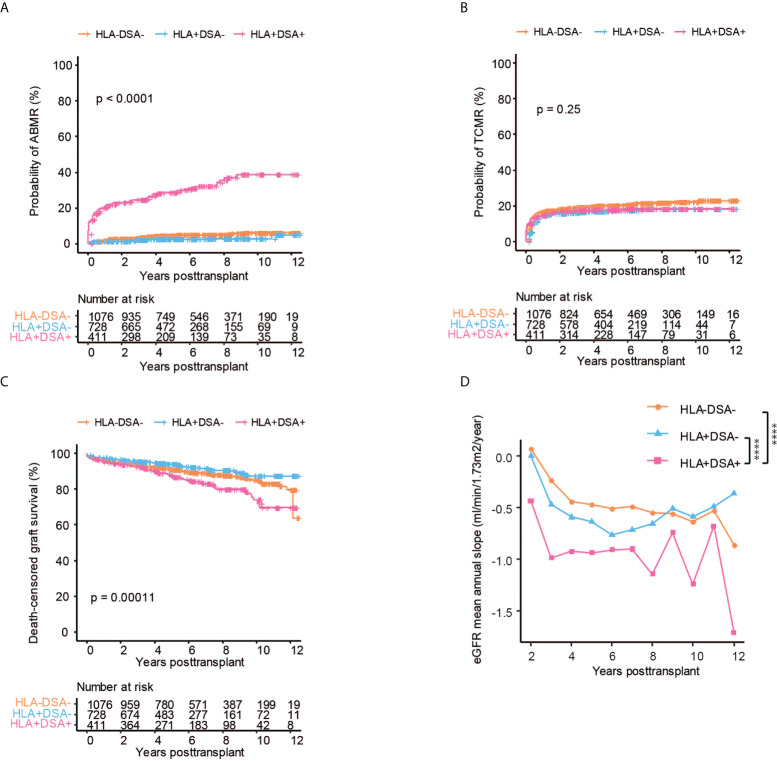
The presence of HLA-DSA is associated with an increased risk of ABMR, graft loss, and loss of kidney function. Cumulative incidence of ABMR **(A)**, TCMR **(B)**, death-censored graft survival **(C)**, and the collective mean annual slope of eGFR **(D)** in patients without anti-HLA antibodies and without DSA (HLA-DSA-), with anti-HLA antibodies but without DSA (HLA+DSA-) and in patients with DSA (HLA+DSA+) respectively. Log-rank test was used to test p value of the Kaplan-Meier survival curves for **(A–C)**. Two-way ANOVA analysis with Sidak’s multiple comparisons as a *post hoc* test was used for **(D)** to assess p values; ****p < 0.0001.

Pre-transplant DSA significantly increased the risk of ABMR both as compared to patients without anti-HLA antibodies as well as to patients with anti-HLA antibodies but without DSA (**Figure 2A**). In line with previous data, there was no significant difference in the risk of ABMR between patients with anti-HLA antibodies and lack of DSA as compared to patients without anti-HLA antibodies (18) (**Figure 2A**). We found no difference in the risk of TCMR between any of the investigated subgroups (**Figure 2B**). DSA also significantly affected death censored graft survival, with a 70% graft survival at 10 years as compared to 90% in patients without DSA (**Figure 2C**). The presence of DSA did not significantly affect patient survival in our cohort, even though there was a trend towards worse survival patients with DSA and in patients with ABMR (**Supplementary Figures 1A, B**). The mean annual eGFR slope decline, which is a marker for accelerated decline of graft function in kidney transplantation, was also significantly impacted by the presence of DSA (19, 20) (**Figure 2D**). The eGFR slope trajectories are highly individual, and the mean difference between the subgroups at later time points is affected by graft loss and follow-up time, which influences the analyses (**Supplementary Figure 1C**). The mean total eGFR slope calculated over the complete follow-up (which reduces the effect of graft loss but does not capture temporal dynamics) showed a clear trend towards accelerated eGFR decline in patients with DSA, but this did not reach statistical significance (**Supplementary Figure 1D**). In summary, DSA but not non-DSA anti-HLA antibodies significantly increases the risk for ABMR, accelerated decline of graft function and graft loss.

### Pre-transplant DSA directed against HLA Class II antigens is coupled to decreased graft survival

**Figure 3:**
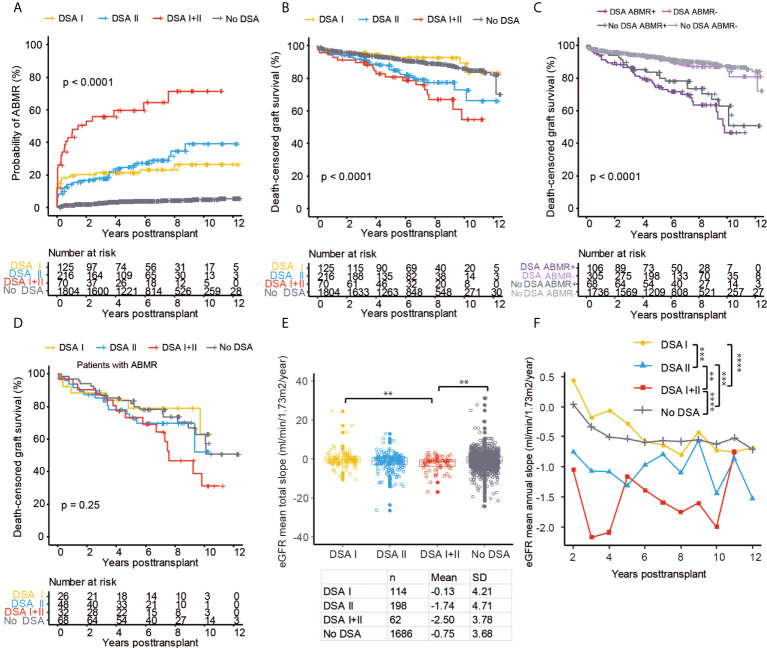
Pre-transplant DSA directed against HLA Class II antigens showed significantly worse outcomes after kidney transplantation. Cumulative incidence of ABMR **(A)** and death-censored graft survival **(B)** in the patient groups with DSA directed against HLA Class I (DSA I), HLA Class II (DSA II), or a combination of Class I and Class II (DSA I+II) and in patients with no DSA. **(C)** Cumulative incidence of death-censored graft survival in the cohort stratified into groups based on the presence or absence of DSA and ABMR. **(D)** Death-censored graft survival in patients with ABMR stratified into DSA I, DSA II, DSA I+II, and no DSA groups. The collective mean total slope **(E)** and mean annual slope **(F)** of eGFR in the DSA I, DSA II, DSA I+II, and no DSA groups. Log-rank test was used to test p value of the Kaplan-Meier survival curves for **(A–D)**. One-way ANOVA followed by Dunn’s *post hoc* test for **(E)** and two-way ANOVA analysis with Sidak’s multiple comparisons as a *post hoc* test were used for **(F)** to assess p values; **p < 0.01, ***p < 0.001, ****p < 0.0001.

Previous studies have shown conflicting data regarding the possible different impact of pre-transplant DSA directed against HLA Class I (DSA I) and Class II (DSA II) antigens (21–24). However, several studies have suggested that DSA II is associated with an inferior outcome (23, 25). In our study, the risk of ABMR was similarly increased with DSA I and DSA II when compared to the risk in patients without DSA (**Figure 3A**). Patients with combined DSA I + DSA II had an even higher risk for ABMR development, with >60% having been diagnosed with ABMR at 4 years after transplantation (**Figure 3A**) but no distinct difference in TCMR risk (**Supplementary Figure 2A**). Long-term graft survival was also significantly worse in both patients with DSA II and with a combination of DSA I + DSA II (**Figure 3B**). Interestingly, the presence of only DSA I did not significantly affect long-term graft survival, even though it was associated with increased risk of ABMR (**Figure 3B**). When we stratified our cohort based on the presence of pre-transplant DSA and ABMR, we could show that patients with DSA that did not develop ABMR had comparable long-term graft survival to patients without DSA and without ABMR (**Figure 3C**). Patients without pre-transplant DSA that developed ABMR, likely consisting largely of patients with post-transplant *de novo* DSA development, had comparable long-term graft outcomes to patients with DSA and ABMR (**Figure 3C**). In patients with ABMR and DSA, we could observe a trend towards worse graft survival in patients with DSA I + II as compared to patients with DSA I (**Figure 3D**). In concurrence with our previous data, the presence of DSA I +DSA II was associated with significantly worse mean total eGFR slope both as compared to DSA I and patients without DSA (**Figure 3E**). Analyses of the mean annual eGFR slope also showed a significantly accelerated decline of graft function in patients with DSA II or with DSA I + II as compared to patients with DSA I or without DSA (**Figure 3F**; **Supplementary Figure 2B**). In summary, both Class I and Class II directed DSA are associated with increased risk for ABMR but only patients with Class II DSA show evidence of accelerated decline of graft function as well as increased risk of graft loss.

### Pre-transplant DSA MFI significantly affects transplant outcome

**Figure 4:**
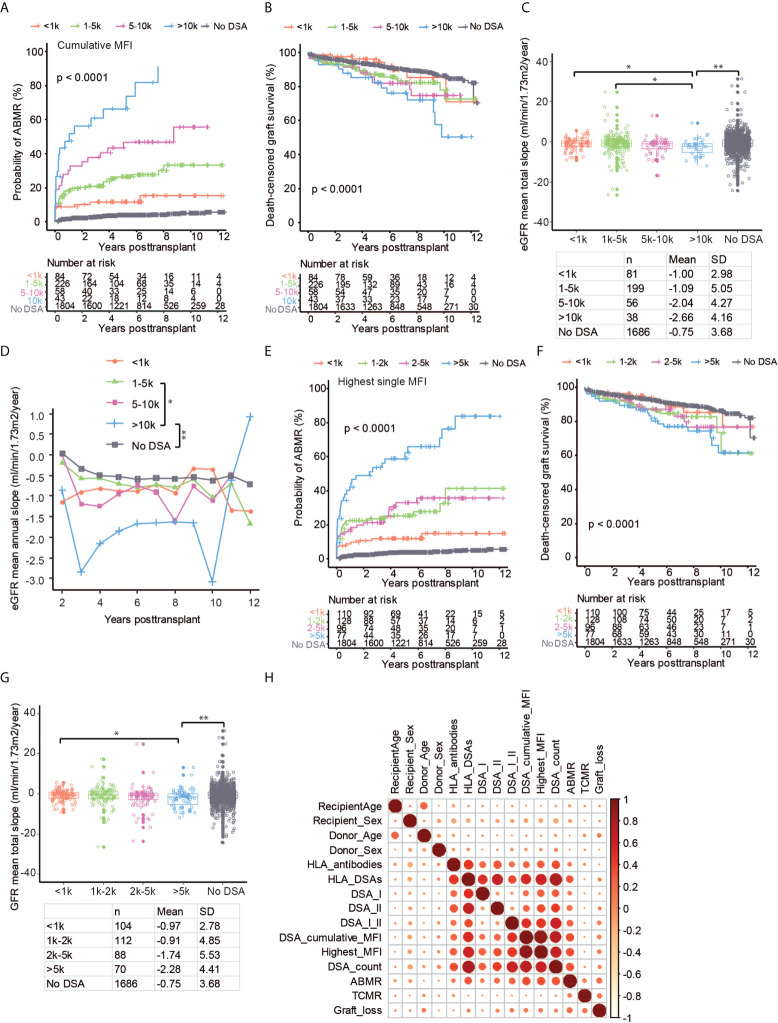
The MFI of the pre-transplant DSA has a large impact on kidney transplant outcome. Cumulative incidence of ABMR **(A)**, death-censored graft survival **(B)**, the collective mean total slope **(C)**, and mean annual slope of eGFR **(D)** in DSA positive patients stratified on the total cumulative MFI into <1k, 1k-5k, 5k-10k, >10k MFI as well as patients with no DSA. Cumulative incidence of ABMR **(E)**, death-censored graft survival **(F)**, and the collective mean total slope **(G)** in DSA positive patients stratified on the single highest MFI of the detected DSA into groups with <1k, 1k-2k, 2k-5k >5k MFI as well as patients with no DSA. **(H)** Correlation heat map of kidney transplantation outcome (ABMR, TCMR, and graft loss) with DSA and clinical variables among all the patients. Dot sizes and colors correspond to the Spearman’s correlation coefficient. Log-rank test was used to test p value of the Kaplan-Meier survival curves for **(A, B)** and **(E, F)**. One-way ANOVA followed by Dunn’s *post hoc* test for **(C)** and **(G)** and two-way ANOVA analysis with Sidak’s multiple comparisons as a *post hoc* test were used for **(D)** to assess p values; *p < 0.05, **p < 0.01.

We next sought to investigate the impact of DSA MFI on graft outcome by measuring cumulative MFI of the detected DSA or the highest single DSA MFI. Cumulative DSA MFI had a striking impact on the risk of ABMR (**Figure 4A**). Interestingly, DSA with a MFI between 500-1000 were also associated with a significantly increased risk of ABMR as compared to patients without DSA (**Figure 4A**). The increased risk of ABMR for DSA MFI<1000 did not, however, markedly impact graft survival as compared to patients without pre-transplant DSA (**Figure 4B**). Pre-transplant DSA with MFI >1000 significantly decreased graft survival, with the worst outcome observed for patients with cumulative DSA MFI >5000 (**Figure 4B**). Graft function as measured by mean total eGFR slope or by investigating mean annual eGFR slope was also affected by the MFI of pre-transplant DSA with the largest loss of eGFR experienced by patients with high cumulative MFI DSA (**Figures 4C, D**; **Supplementary Figure 3A**). For highest single DSA MFI, the risk of ABMR was also significantly increased in all of the stratified groups with DSA (**Figure 4E**). However, only a highest DSA MFI of >1000 MFI was associated with markedly worse long-term graft survival (**Figure 4F**). Single DSA with a MFI of >2000 showed a trend towards faster decline of graft function as measured by mean total eGFR slope (**Figure 4G**). This indicates that both cumulative DSA MFI and highest single DSA MFI are associated with graft outcome but that cumulative DSA MFI might more accurately capture the immunological risk. In order to illustrate the association between DSA, MFI, graft outcome as well as recipient and donor age and sex, we constructed a correlation matrix (**Figure 4H**). Most of the investigated DSA parameters were highly correlated with ABMR, whereas TCMR was not associated with any DSA characteristics (**Figure 4H**). As expected, recipient sex was also correlated to anti-HLA antibodies, DSA and ABMR, whereas there was no discernable association between sex and TCMR. In summary both cumulative DSA MFI as well as the single highest DSA MFI are strongly associated with the risk of ABMR, accelerated decline of graft function and graft loss.

### Differential impact of MFI on Class I and Class II DSA

**Figure 5:**
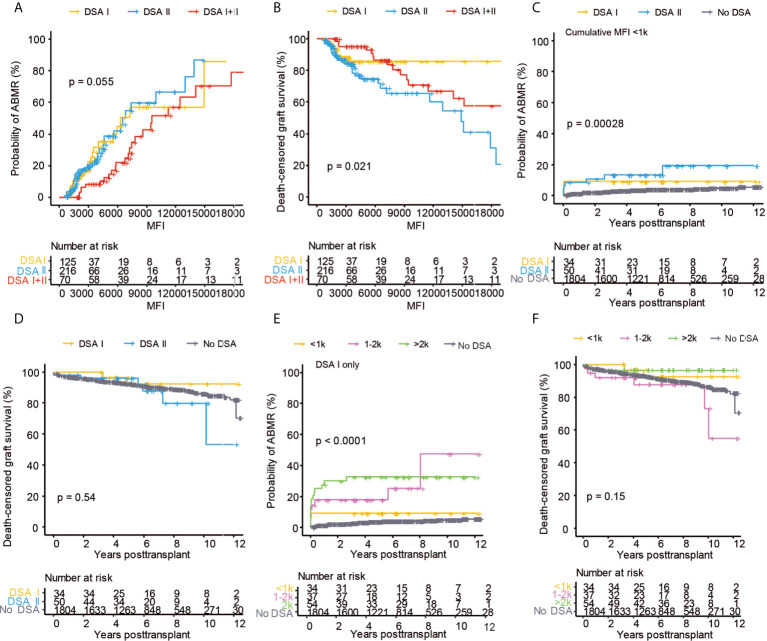
The MFI of pre-transplant DSA directed against HLA class I and II show a different impact on kidney transplant outcome. Cumulative incidence of ABMR **(A)** and death-censored graft survival **(B)** in the DSA I, DSA II, DSA I+II groups with regard to cumulative DSA MFI value. Cumulative incidence of ABMR **(C)** and death-censored graft survival **(D)** in the DSA I and DSA II groups in patients with a cumulative MFI of <1k. Cumulative incidence of ABMR **(E)** and death-censored graft survival **(F)** of patients with only DSA I stratified into groups based on cumulative MFI of <1k, 1k-2k, and >2k. Log-rank test was used to test p value of the Kaplan-Meier survival curves for **(A–F)**.

Based on our finding of the varying incidence of ABMR and graft loss in patients with Class I and Class II DSA, we decided to study these differences in detail. By directly investigating the risk of ABMR and graft loss in relation to DSA Class and MFI, it was clear that increased MFI was related to both the risk of ABMR and graft loss in all of the DSA Class combinations (**Figures 5A, B**). The risk of graft loss for patients with DSA I did not, however, dramatically increase at higher MFI (>7000), but this should be interpreted with caution due to the very limited number of patients that were transplanted with the presences of a DSA I with MFI >7000 (**Figure 5B**). To further investigate the impact of DSA with low MFI, we stratified patients with a cumulative DSA MFI of <1000 (n=84) into patients with isolated DSA I and DSA II and subsequently investigated the impact on AMBR and graft loss. The probability of AMBR was significantly increased in patients with DSA II whereas DSA I did not markedly affect long-term ABMR risk (**Figure 5C**). Graft survival was not significantly lower in patients with a DSA MFI<1000, however there was a clear trend towards worse long-term graft survival in patients with DSA II whereas patients with DSA I MFI<1000 did not show worse graft survival when compared to patients without DSA (**Figure 5D**). When we instead analyzed patients with a cumulative DSA MFI >1000, we observed a comparable risk of ABMR for patients with DSA I and DSA II but only DSA II (and DSA I +II) was associated with significantly worse graft survival (**Supplementary Figures 4A, B**). This prompted us to investigate the relationship between DSA I and MFI further by stratifying patients with DSA I into groups with MFI <1000, 1000-2000, and >2000. There was a significant increased risk of AMBR in patients with DSA I MFI >1000 but this was not associated with a significantly increased graft loss (**Figures 5E, F**). We also analyzed the impact of low MFI DSA on eGFR slope in the same subgroups and here we could only find a slight trend for accelerated decline in kidney function in patients with DSA II MFI <1000 as compared to patients with DSA I (**Supplementary Figures 4C, D**). The analysis of the different low MFI DSA I strata did not show any consistent trend (**Supplementary Figures 4E, F**). In summary, our data shows an increase for ABMR associated with low MFI Class II DSA and suggests that the risk assessment of low MFI DSA could be differentially addressed based on the target antigen HLA Class of the detected DSA.

### Impact of DSA directed at different HLA loci

**Figure 6:**
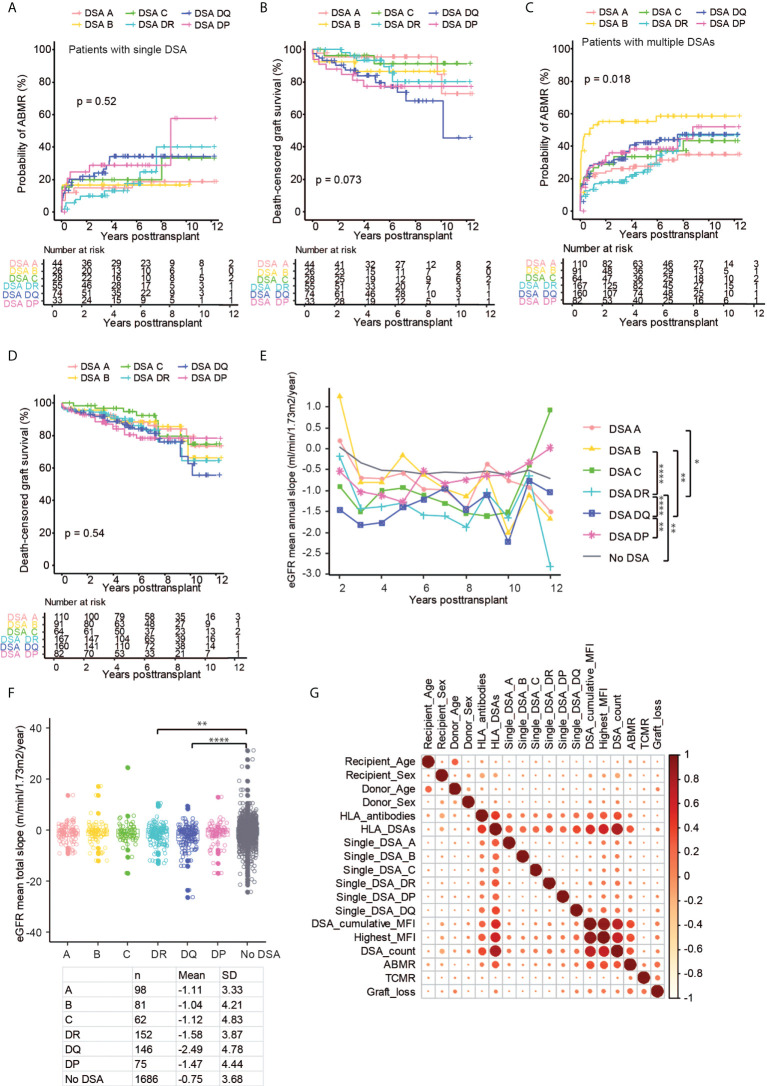
The impact of the HLA target loci on kidney transplant outcomes in patients with single and multiple DSA. Cumulative incidence of ABMR **(A)** and death-censored graft survival **(B)** in patients with a single DSA directed against HLA-A (DSA A), HLA-B (DSA B), HLA-C (DSA C), HLA-DR (DSA DR), HLA-DQ (DSA DQ) and HLA-DP (DSA DP). Cumulative incidence of ABMR **(C)**, death-censored graft survival **(D)**, the collective mean total slope **(E)**, and mean annual slope of eGFR **(F)** in the patients with combinations of DSA, denoted as mentioned previously. **(G)** Correlation heat map of kidney transplantation outcome (ABMR, TCMR, and graft loss) DSA characteristics and clinical variables among all investigated patients. Log-rank test was used to test p value of the Kaplan-Meier survival curves for **(A–D)**. One-way ANOVA followed by Dunn’s *post hoc* test for **(E)** and two-way ANOVA analysis with Sidak’s multiple comparisons as a *post hoc* test were used for **(F)** to assess p values; *p < 0.05, **p < 0.01, ****p < 0.0001.

**Figure 7:**
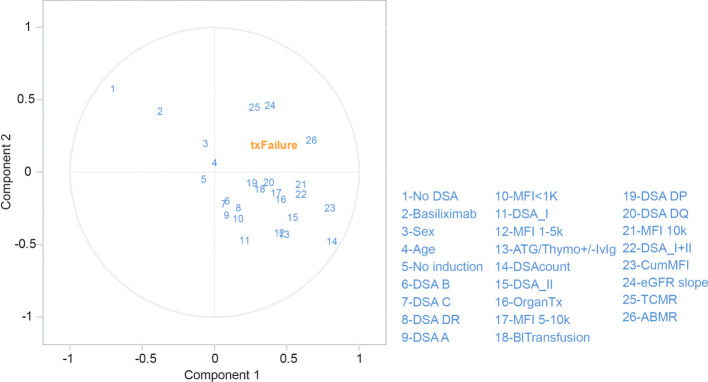
Partial least squares (PLS) regression biplot for the first two components in graft loss. Correlation shown between graft loss (txFailure) and the risk factors (in blue numbers). The first two axes which correspond to PLS components 1 and 2 are shown. The distance between the individual risk factors and the center indicates the strength of the correlation with each component and their alignments represent the correlation they contribute to the variation explained by each component within the model. (OrganTx, Organ Transplantation; BlTransfusion, Blood Transfusion).

The large group of patients with DSA within our cohort made it possible to study the impact of DSA directed at individual HLA loci. Previous studies have shown conflicting evidence on the impact of DSA directed at HLA-C and HLA-DP, and there have been reports suggesting a worse outcome in the setting of pre-transplant HLA-DQ DSA (23, 25–30). In order to study this in an independent way, we selected patients with a single pre-transplant DSA directed at only one HLA locus (n=260) and stratified them on DSA locus. The MFI for the individual locus directed DSA in patients with either single DSA or multiple DSA showed no significant difference (**Supplementary Figures 5A, B**). The risk of ABMR was increased for DSA against all HLA loci, and there was a trend towards a relatively higher risk of ABMR in patients with DSA directed against HLA-DP and HLA-DQ at early time-points after transplantation (**Figure 6A**). At later time-points, DSA against HLA-DR as well as somewhat surprisingly HLA-C, even though the latter is based on fewer observations, were also associated with a higher risk for ABMR (**Figure 6A**). HLA-C DSA were, however, not significantly associated with decreased graft survival in our cohort, and only patients with DSA directed at Class II antigens showed a decreased graft survival (**Figure 6B**). Surprisingly, DSA directed at HLA DP were associated with a marked decline in graft survival that was comparable to the diminished survival seen in the setting of HLA-DQ DSA during the first 6 years post-transplant (**Figure 6B**). In order to expand our data, we also included patients with DSA against multiple loci (**Supplementary Table 2**). We here considered each locus specific DSA as a separate event with a corresponding transplant outcome, which led to the analysis of 674 separate locus specific DSA events. The risk of ABMR was again increased for all DSA events, and DSA against Class II tended to have a higher risk at later time-points (**Figure 6C**). Surprisingly, the highest risk of ABMR in this analysis was observed for DSA events directed at HLA-B, but this trend was driven by patients with DSA against HLA-B in combination with DSA directed at Class II antigens (**Supplementary Figure 5C**). A similar analysis for graft survival also showed a trend towards worse survival in patients with DSA directed at HLA-DR, DQ, and DP even though the signal was much less clear as compared to our analyses of single DSA patients (**Figure 6D**). DSA against HLA-DQ were coupled to the largest early decrease in mean annual eGFR slope, whereas DSA against HLA-A and HLA-B did not show a significant difference to patients without DSA (**Figure 6E**). When the mean total eGFR slope was analyzed, there was a clear trend for a more rapid decline of eGFR in patients with Class II DSA (**Figure 6F**). Indeed both patients with DSA directed at HLA-DQ and HLA-DR showed a significantly faster decline in eGFR as compared to patients without DSA (**Figure 6F**). To show the association between DSA HLA locus target, MFI, graft outcome as well as recipient and donor age and sex, we constructed a correlation matrix (**Figure 6G**). Both ABMR and graft loss were strongly correlated with the presence of DSA, DSA MFI, and DSA count, and the strongest association with ABMR and graft loss was seen for DSA targeting HLA-DQ (**Figure 6G**). A univariate cox regression analyses also showed a similar pattern with regards to immunological risk factors in relation to graft loss (**Supplementary Table 3**). We next used a partial least squares (PLS) regression model to illustrate the relationship between graft loss and different risk factors listed in **Supplementary Table 3** in a multivariate model (**Figure 7**). ABMR, TCMR and eGFR decline fell in the same quadrant as graft loss, indicating that they were more closely aligned together and influenced by the similar risk factors explained by both component 1 and 2. Other indicators such as DSA against HLA-B, HLA-C, HLA-DR, HLA-A, class I and MFI <1K were clustered together centrally indicating a more distant relationship with graft loss. This was in contrast with blood transfusion, previous transplantation, DSA against HLA-DP, HLA-DQ, Class II, MFI 5-10K and MFI>10 that were clustered together on component 1 closer to the graft loss quadrant indicating a more dependent relationship. Consistent with our previous findings (**Figure 2A**), the PLS-regression for ABMR (**Supplementary Figure 6A**) showed that DSA-related factors were positively correlated with the development of ABMR. However, a similar effect was not observed in the development of TCMR (**Supplementary Figure 6B**).

In summary, our analyses of patients with DSA directed at a single loci show comparable risk for ABMR between all HLA loci but increased risk for graft loss was only consistently over several analyses observed in the setting of DSA directed against Class II antigens.

## Discussion

Despite numerous studies that have shown a strong association between the presence of DSA and inferior transplant outcome, several important questions related to the effect of the MFI value, as well as the HLA locus specificity of the DSA have not been conclusively investigated. By investigating 411 DSA positive kidney transplantations within the STCS and comparing them to 1804 transplantations without DSA performed in the same centers during the same period, we were able to perform the to-date largest study of the impact of pre-transplant DSA on long-term transplant outcome in patients with a complete vXM.

As previously shown in smaller studies DSA but not non-DSA anti-HLA antibodies were significantly associated with increased risk for ABMR, accelerated decline of eGFR, and graft loss (18, 31). When the DSA were stratified on their targeted HLA Class, there was a clear difference concerning transplant outcome between Class I and Class II directed DSA. For DSA against Class I, we could observe an increased frequency of ABMR, but this was not associated with a significantly increased graft loss. This was also evident when we examined patients with only single DSA directed at one donor specific HLA antigen. Here, DSA against HLA-A, B, and C were associated with increased risk of ABMR but to a much lesser extent with graft loss. Conversely, DSA against Class II antigens were consistently associated with both increased risk of ABMR and graft loss. There was also no large difference between the different Class II antigens except for a slight trend towards worse long-term graft survival and increased rate of graft function decline as measured by eGFR slope in the setting of DSA that target HLA-DQ, as has been previously suggested (23, 25). Interestingly, DSA against Class II with a cumulative MFI below 1000 were also associated with significantly increased risk of ABMR. There was also a trend towards increased graft loss in these patients that was detectable >6 years after transplantation which strongly argues that the impact of low MFI pre-transplant DSA should be evaluated long-term and that shorter outcome data do not fully capture the impact of this pre-existing donor specific alloimmunity. The MFI of the detected DSA had a large effect on transplant outcome both when cumulative DSA MFI or highest single DSA MFI was considered. When patients were stratified into groups based on their DSA MFI, there was a very clear separation between the groups concerning ABMR, graft loss, and eGFR decline. In the clinical setting, a single DSA MFI value will always be interpreted in the context of the antigen specificity of the detected DSA. As previously discussed, we were not able to observe a significantly increased frequency of graft loss in patients with single DSA directed at Class I antigens. This could primarily be attributed to the fact that a large fraction of Class I DSA were in the 500-2000 MFI range. When we stratified the Class I DSA on MFI, we observed significantly increased risk for ABMR in DSA MFI >1000 but this was again not coupled to a consistent trend for increased graft loss. Our data suggests that different cumulative DSA MFI cut-offs could be used to further optimize vXM algorithms. Here, our suggestion would be to only consider Class I DSA with an MFI >1000, whereas Class II DSA MFI could be taken into account also in the 500-1000 MFI range. Furthermore, the increase in ABMR incidence and graft loss seen with Class I DSA MFI>1000 did not reach statistical significance, which argues that transplantations against Class I DSA with higher MFI could also be considered. We could not find significant differences between DSA directed at the different Class I loci, and our data suggests that they could be associated with similar risk. For the different Class II loci, we were surprised to find that DSA against HLA-DP appeared to be associated with similar early increased risk as DSA towards HLA-DQ. As previously described, there was a clear signal indicating that DSA against HLA-DQ are of particular concern as they show the strongest association with accelerated decline of graft function and graft loss (23, 25). This finding could be influenced by the fact that slightly more transplantations were performed within our cohort with high MFI HLA-DQ directed DSA (**Figure 1D**). We employed a PLS regression model to accurately analyze the relationship of our investigated variables with graft loss in the setting of collinearity of several of the investigated variables. Our analyses confirmed several of our previous findings but also revealed interesting relationships not visible in our univariate analyses. Even though the PLS regression model has many advantages with regards to our investigated data it has not been previously employed in the analysis of transplant immunological risk factors and the results should be interpreted accordingly.

We used two different methods to calculate eGFR decline. The first illustrated the development of the mean eGFR slope over the complete follow-up time by use of annual eGFR calculations (19, 20). The second method evaluated the total eGFR slope for each patient as calculated to the latest follow-up time. The first method has the ability to show how the eGFR slope changes over time after transplantation but is subjected to bias at later time points, especially in subgroups with a high amount of graft loss, as these patients are no longer represented within the mean. Our second method eliminates this problem but does not allow for a detailed picture of the change in eGFR over the study period. Our single patient plots also illustrate the extreme variability in graft function within the different groups, which makes the comparison between different subgroups challenging. Despite these caveats, we were able to show a significant difference in the eGFR trajectories of several different subgroups, which further underscores the crucial impact of pre-transplant DSA on the continuous loss of graft function post-transplantation.

Our study has several limitations related to the multicenter design and long inclusion period, including differences in induction and maintenance immunosuppressive therapies at the different centers, as well as related to evaluation of SAB results and individual procedures for the diagnosis and therapy of rejection. Development of *de novo* DSA or antibody kinetics of pre-transplant DSA post transplantation is not captured in the STCS database and we are therefore unable to assess the effect of these important markers on the outcome of transplantation.

In summary, we present data on the impact of pre-transplant DSA on transplant outcome in 411 DSA positive transplantations. Our study is the largest to date with a complete vXM and provides important data that can be used to further improve vXM algorithms by individualizing immunological risk associated with DSA MFI and HLA antigen target.

## Data availability statement

The original contributions presented in the study are included in the article/**Supplementary Material**. Further inquiries can be directed to the corresponding author.

## Ethics statement

The studies involving human participants were reviewed and approved by Cantonal Ethics Committee of Zurich (BASEC-Nr.2021-0083). The patients/participants provided their written informed consent to participate in this study.

## Author contributions

LF and YD collected and analyzed data and wrote the manuscript. CW, OR, JV, SF-L, DG, MG, IB, UW, DS, TS, and SS collected data and critically reviewed the manuscript. JN designed the research, collected and analyzed the data, and wrote the manuscript. All authors reviewed and approved the final version of the manuscript.

## Funding

The Swiss Transplant Cohort Study is supported by the Swiss National Science Foundation (https://www.snf.ch), Unimedsuisse (https://www.unimedsuisse.ch) and the Transplant Centers.

## Acknowledgments

This study has been conducted in the framework of the Swiss Transplant Cohort Study, supported by the Swiss National Science Foundation and the Swiss University Hospitals (G15) and transplant centers. The members of the Swiss Transplant Cohort Study are: Patrizia Amico, Andres Axel, John David Aubert, Vanessa Banz, Beckmann Sonja, Guido Beldi, Christoph Berger, Ekaterine Berishvili, Isabelle Binet, Pierre-Yves Bochud, Sanda Branca, Heiner Bucher, Thierry Carrel, Emmanuelle Catana, Yves Chalandon, Sabina De Geest, Olivier De Rougemont, Michael Dickenmann, Joëlle Lynn Dreifuss, Michel Duchosal, Thomas Fehr, Sylvie Ferrari-Lacraz, Nicola Franscini, Christian Garzoni, Paola Gasche Soccal, Christophe Gaudet, Déla Golshayan, Nicolas Goossens, Karine Hadaya, Jörg Halter, Dominik Heim, Christoph Hess, Sven Hillinger, Hans Hirsch, Patricia Hirt, Günther Hofbauer, Uyen Huynh-Do, Franz Immer, Michael Koller (Head of the data center), Mirjam. Laager, Bettina Laesser, Roger Lehmann, Alexander Leichtle, Christian Lovis, Oriol Manuel, Hans-Peter Marti, Pierre Yves Martin, Michele Martinelli, Valérie McLin, Katell Mellac, Aurelia Mercay, Karin Mettler, Nicolas Mueller (Chairman Scientific Committee), Antonia Müller, Thomas Müller, Ulrike Müller-Arndt, Beat Müllhaupt, Mirjam Nägeli, Graziano Oldani, Manuel Pascual (Executive office), Klara Posfay-Barbe, Juliane Rick, Anne Rosselet, Simona Rossi, Silvia Rothlin, Frank Ruschitzka, Urs Schanz, Stefan Schaub, Aurelia Schnyder, Macé Schuurmans, Thierry Sengstag, Federico Simonetta, Katharina Staufer, Susanne Stampf, Jürg Steiger (Head, Excecutive office), Guido Stirniman, Ueli Stürzinger, Christian Van Delden (Executive office), Jean-Pierre Venetz, Jean Villard, Julien Vionnet,Madeleine Wick (STCS coordinator), Markus Wilhlem, Patrick Yerly.

## Conflict of interest

The authors declare that the research was conducted in the absence of any commercial or financial relationships that could be construed as a potential conflict of interest.

## Publisher’s note

All claims expressed in this article are solely those of the authors and do not necessarily represent those of their affiliated organizations, or those of the publisher, the editors and the reviewers. Any product that may be evaluated in this article, or claim that may be made by its manufacturer, is not guaranteed or endorsed by the publisher.

## Supplementary material

Supplementary Figure 1 The influence of HLA-DSA on kidney transplantation outcome. **(A)** Overall patient survival in patients with and without DSA. **(B)** Overall survival in patients that developed rejection (ABMR or TCMR) and in patients without rejection. The individual eGFR **(C)** trajectory plots in patients without anti-HLA antibodies (HLA-DSA-), with anti-HLA antibodies but without DSA (HLA+DSA-), and in patients with DSA (HLA+DSA+). **(D)** The collective mean total slope of eGFR in patients with HLA-DSA-, HLA+DSA- and HLA+DSA+, respectively. Log-rank test was used to test p value of the Kaplan-Meier survival curves for **(A**, **B)**. One-way ANOVA (Kruskal-Wallis test) followed by Dunn’s *post hoc* test for **(D)**.

Supplementary Figure 2 The impact of DSA directed against Class I or Class II. Cumulative incidence of TCMR **(A)** and the individual eGFR trajectory **(B)** in in the patient groups with DSA directed against HLA Class I (DSA I), HLA Class II (DSA II), or a combination of Class I and Class II (DSA I+II) and in patients with no DSA. Log-rank test was used to test p value of the Kaplan-Meier survival curves for **(A)**.

Supplementary Figure 3 The impact of MFI on the eGFR slope. **(A)** The individual eGFR trajectories in the patient groups with a cumulative DSA MFI of <1k, 1k-5k, 5k-10k, >10k, and no DSA. **(B)** The collective mean annual slope of eGFR in groups stratified on the single highest MFI of the detected DSA into <1k, 1k-2k, 2k-5k >5k, and no DSA groups. Two-way ANOVA analysis with Sidak’s multiple comparisons as a *post hoc* test were used for **(B)** to assess p values; *p<0.05, **p<0.01,

Supplementary Figure 4 Different DSA Classes differentially affect the outcome of kidney transplantation. Cumulative incidence of ABMR **(A)** and death-censored graft survival **(B)** in the DSA I, DSA II, DSA I+II groups with a cumulative MFI value >1k. The collective mean total slope of eGFR **(C)** and mean annual slope **(D)** in the DSA I and DSA II groups with cumulative MFI <1k. The collective mean total slope of eGFR **(E)** and mean annual slope **(F)** of patients with only DSA I in groups stratified on MFI into <1k, 1k-2k, and >2k. Log-rank test was used to test p value of the Kaplan-Meier survival curves for **(A, B)**. One-way ANOVA followed by Dunn’s *post hoc* test for **(C)** and **(E)**, two-way ANOVA analysis with Sidak’s multiple comparisons as a *post hoc* test were used for **(D)** and **(F)** to assess p values; *p<0.05.

Supplementary Figure 5 DSA MFI overview and cumulative incidence of ABMR DSA combinations containing DSA against HLA-B. Violin plots of all detected DSA grouped into target HLA loci for patients with a single DSA **(A)** or with multiple DSA **(B)**. **(C)** Cumulative incidence of ABMR in the presence of DSA directed against HLA-B only (DSA B only), or in patients with DSA directed at HLA-B + another Class I loci (DSA B + DSA I) and in patients with DSA directed at HLA-B + another Class II locus or a combination of additional Class I and Class II loci (DSA B + DSA II/I+II). One-way ANOVA (Kruskal-Wallis test) followed by Dunn’s *post hoc* test for **(A, B)** to assess the p value. Log-rank test was used to test p value of the Kaplan-Meier survival curves for **(C)**.

Supplementary Figure 6 Partial least squares (PLS) regression biplot for the first two components in ABMR and TCMR. Correlation shown between ABMR **(A)**, TCMR **(B)** and the risk factors (in blue numbers). The first two axes which correspond to PLS components 1 and 2 are shown. The distance between the individual risk factors and the center indicates the strength of the correlation with each component and their alignments represent the correlation they contribute to the variation explained by each component. (OrganTx, Organ Transplantation; BlTransfusion, Blood Transfusion).
