# Supplementary material for: The impact of pre-transplant donor specific antibodies on the outcome of kidney transplantation – Data from the Swiss transplant cohort study
Source: Front Immunol. 2022 Sep 21;13:1005790. doi: 10.3389/fimmu.2022.1005790 (PMC9532952; doi:10.3389/fimmu.2022.1005790)
Supplement: Supplementary file 1 [file Table_1.docx]

| **Supplementary Table 1. Overview of all the DSA combinations** | | | | |  |
| --- | --- | --- | --- | --- | --- |
| Number | DSA combinations | Count, percentage | Number | DSA combinations | Count, percentage |
| 1 | A+A | n=3, 2% | 23 | B+C+DP | n=1, 1% |
| 2 | A+B | n=8, 5% | 24 | B+C+DQ | n=1, 1% |
| 3 | A+B+C | n=4, 3% | 25 | B+C+DR | n=2, 1% |
| 4 | A+C | n=1, 1% | 26 | B+C+DR+DP | n=1, 1% |
| 5 | B+B | n=4, 3% | 27 | B+C+DR+DP+DQ | n=1, 1% |
| 6 | B+C | n=3, 2% | 28 | B+DP | n=2, 1% |
| 7 | C+C | n=4, 3% | 29 | B+DQ | n=4, 3% |
| 8 | A+B+C+DR | n=1, 1% | 30 | B+DR | n=2, 1% |
| 9 | A+B+C+DR+DQ | n=1, 1% | 31 | B+DR+DP+DQ | n=1, 1% |
| 10 | A+B+DP | n=2, 1% | 32 | B+DR+DQ | n=5, 3% |
| 11 | A+B+DQ | n=6, 4% | 33 | C+DP | n=2, 1% |
| 12 | A+B+DR | n=2, 1% | 34 | C+DP+DQ | n=1, 1% |
| 13 | A+B+DR+DQ | n=2, 1% | 35 | C+DQ | n=3, 2% |
| 14 | A+C+DR | n=1, 1% | 36 | C+DR | n=1, 1% |
| 15 | A+C+DR+DQ | n=1, 1% | 37 | DP+DP | n=4, 3% |
| 16 | A+DP | n=4, 3% | 38 | DP+DQ | n=6, 4% |
| 17 | A+DP+DQ | n=1, 1% | 39 | DP+DR | n=2, 1% |
| 18 | A+DQ | n=8, 5% | 40 | DQ+DQ | n=5, 3% |
| 19 | A+DR | n=7, 5% | 41 | DR+DP | n=6, 4% |
| 20 | A+DR+DP | n=1, 1% | 42 | DR+DP+DQ | n=1, 1% |
| 21 | A+DR+DP+DQ | n=3, 2% | 43 | DR+DQ | n=17, 12% |
| 22 | A+DR+DQ | n=3, 2% | 44 | DR+DR | n=13, 9% |
