# Supplementary material for: The impact of pre-transplant donor specific antibodies on the outcome of kidney transplantation – Data from the Swiss transplant cohort study
Source: Front Immunol. 2022 Sep 21;13:1005790. doi: 10.3389/fimmu.2022.1005790 (PMC9532952; doi:10.3389/fimmu.2022.1005790)
Supplement: Supplementary file 2 [file Table_2.docx]

| **Supplementary Table 2. DSA directed at individual HLA loci in relation to the development of ABMR and graft loss** | | | | | |
| --- | --- | --- | --- | --- | --- |
| **HLA-DSA class I** | **Associated with ABMR (%) (n of total)** | **Associated with gf loss (%) (n of total)** | **HLA-DSA class II** | **Associated with ABMR (%) (n of total)** | **Associated with gf loss (%) (n of total)** |
| A1 | 54 (7 of 13) | 31 (4 of 13) | DP1 | 0 (0 of 7) | 0 (0 of 7) |
| A11 | 25 (2 of 8) | 0 (0 of 8) | DP10 | 60 (3 of 5) | 60 (3 of 5) |
| A2 | 17 (3 of 18) | 11 (2 of 18) | DP11 | 60 (3 of 5) | 0 (0 of 5) |
| A23 | 0 (0 of 2) | 0 (0 of 2) | DP14 | 40 (2 of 5) | 20 (1 of 5) |
| A24 | 31 (4 of 13) | 16 (2 of 13) | DP17 | 40 (2 of 5) | 20 (1 of 5) |
| A25 | 0 (0 of 3) | 0 (0 of 3) | DP19 | 0 (0 of 1) | 0 (0 of 1) |
| A26 | 38 (3 of 8) | 25 (2 of 8) | DP2 | 0 (0 of 4) | 0 (0 of 4) |
| A29 | 50 (3 of 6) | 17 (1 of 6) | DP3 | 39 (7 of 18) | 6 (1 of 18) |
| A3 | 29 (2 of 7) | 14 (1 of 7) | DP4 | 20 (4 of 20) | 15 (3 of 20) |
| A30 | 0 (0 of 3) | 0 (0 of 3) | DP5 | 50 (3 of 6) | 17 (1 of 6) |
| A31 | 14 (1 of 7) | 14 (1 of 7) | DP6 | 0 (0 of 2) | 50 (1 of 2) |
| A32 | 20 (2 of 10) | 0 (0 of 10) | DP9 | 25 (1 of 4) | 0 (0 of 4) |
| A33 | 0 (0 of 2) | 0 (0 of 2) | DQ2 | 26 (8 of 31) | 6 (2 of 31) |
| A68 | 11 (1 of 9) | 22 (2 of 9) | DQ4 | 50 (2 of 4) | 13 (1 of 4) |
| A69 | 0 (0 of 1) | 0 (0 of 1) | DQ5 | 36 (8 of 22) | 18 (4 of 22) |
| B13 | 25 (1 of 4) | 0 (0 of 4) | DQ6 | 42 (13 of 31) | 16 (5 of 31) |
| B18 | 33 (1 of 3) | 33 (1 of 3) | DQ7 | 19 (8 of 42) | 19 (8 of 42) |
| B27 | 0 (0 of 3) | 33 (1 of 3) | DQ8 | 42 (8 of 19) | 37 (7 of 19) |
| B35 | 40 (4 of 10) | 0 (0 of 10) | DQ9 | 40 (4 of 10) | 10 (1 of 10) |
| B37 | 50 (2 of 4) | 0 (0 of 4) | DR1 | 44 (4 of 9) | 0 (0 of 9) |
| B38 | 100 (3 of 3) | 0 (0 of 3) | DR10 | 0 (0 of 1) | 0 (0 of 1) |
| B39 | 0 (0 of 1) | 0 (0 of 1) | DR11 | 20 (2 of 10) | 30 (3 of 10) |
| B41 | 0 (0 of 1) | 0 (0 of 1) | DR12 | 0 (0 of 2) | 0 (0 of 2) |
| B44 | 40 (4 of 10) | 10 (1 of 10) | DR13 | 20 (2 of 10) | 30 (3 of 10) |
| B45 | 0 (0 of 1) | 0 (0 of 1) | DR14 | 33 (1 of 3) | 66 (2 of 3) |
| B50 | 100 (3 of 3) | 33 (1 of 3) | DR15 | 20 (2 of 10) | 20 (2 of 10) |
| B51 | 29 (2 of 7) | 14 (1 of 7) | DR16 | 0 (0 of 4) | 0 (0 of 4) |
| B55 | 33 (1 of 3) | 0 (0 of 3) | DR17 | 0 (0 of 4) | 0 (0 of 4) |
| B57 | 56 (5 of 9) | 11 (1 of 9) | DR18 | 0 (0 of 1) | 0 (0 of 1) |
| B60 | 50 (4 of 8) | 13 (1 of 8) | DR4 | 37 (9 of 26) | 15 (4 of 26) |
| B61 | 100 (1 of 1) | 0 (0 of 1) | DR51 | 0 (0 of 7) | 14 (1 of 7) |
| B62 | 43 (3 of 7) | 14 (1 of 7) | DR52 | 14 (4 of 29) | 10 (3 of 29) |
| B63 | 100 (1 of 1) | 0 (0 of 1) | DR53 | 34 (10 of 29) | 10 (3 of 29) |
| B7 | 25 (2 of 8) | 13 (1 of 8) | DR7 | 30 (6 of 20) | 10 (2 of 20) |
| B73 | 100 (1 of 1) | 0 (0 of 1) | DR8 | 0 (0 of 1) | 100 (1 of 1) |
| B8 | 50 (1 of 2) | 0 (0 of 2) | DR9 | 0 (0 of 1) | 0 (0 of 1) |
| Cw1 | 29 (2 of 7) | 14 (1 of 7) |  |  |  |
| Cw10 | 20 (1 of 5) | 0 (0 of 5) |  |  |  |
| Cw12 | 50 (1 of 2) | 50 (1 of 2) |  |  |  |
| Cw15 | 0 (0 of 6) | 0 (0 of 6) |  |  |  |
| Cw16 | 50 (1 of 2) | 0 (0 of 2) |  |  |  |
| Cw17 | 66 (2 of 3) | 33 (1 of 3) |  |  |  |
| Cw2 | 100 (1 of 1) | 0 (0 of 1) |  |  |  |
| Cw4 | 13 (1 of 8) | 13 (1 of 8) |  |  |  |
| Cw5 | 40 (2 of 5) | 0 (0 of 5) |  |  |  |
| Cw6 | 25 (3 of 12) | 17 (2 of 12) |  |  |  |
| Cw7 | 40 (4 of 10) | 10 (1 of 10) |  |  |  |
| Cw8 | 0 (0 of 2) | 0 (0 of 2) |  |  |  |
| Cw9 | 0 (0 of 1) | 0 (0 of 1) |  |  |  |
