# Supplementary material for: The impact of pre-transplant donor specific antibodies on the outcome of kidney transplantation – Data from the Swiss transplant cohort study
Source: Front Immunol. 2022 Sep 21;13:1005790. doi: 10.3389/fimmu.2022.1005790 (PMC9532952; doi:10.3389/fimmu.2022.1005790)
Supplement: Supplementary file 3 [file Table_3.docx]

| **Supplementary Table 3. Univariate analysis to identify potential predictors of graft loss** | | | | |
| --- | --- | --- | --- | --- |
|  |  | Univariate analysis | |  |
| Parameter | No. of Event | HR | 95% CI | p value |
| Recipient age | 2215 | 1.010 | 0.999-1.019 | 0.070 |
| Recipient gender | 2215 | 1.135 | 0.869-1.483 | 0.354 |
| Previously transplanted | 402 | 1.584 | 1.185-2.119 | **0.002** |
| Previous pregnancy (only females) | 175 | 0.723 | 0.406-1.287 | 0.270 |
| Previous blood transfusion | 683 | 1.326 | 1.020-1.724 | **0.035** |
| DSA counts | 411 | 1.218 | 1.083-1.369 | **0.001** |
| DSA class |  |  |  |  |
| No DSA | 1804 | Reference | | |
| DSA I | 125 | 0.782 | 0.414-1.480 | 0.450 |
| DSA II | 216 | 1.984 | 1.385-2.842 | **<0.001** |
| DSA I+II | 70 | 2.910 | 1.812-4.674 | **<0.001** |
| Single DSA |  |  |  |  |
| No DSA | 1804 | Reference | | |
| DSA A | 44 | 0.865 | 0.321-2.332 | 0.774 |
| DSA B | 26 | 1.352 | 0.432-4.235 | 0.604 |
| DSA C | 28 | 0.704 | 0.175-2.839 | 0.622 |
| DSA DR | 55 | 1.306 | 0.579-2.949 | 0.520 |
| DSA DQ | 74 | 2.847 | 1.728-4.690 | **<0.001** |
| DSA DP | 33 | 2.632 | 1.235-5.606 | **0.012** |
| DSA Cummulative MFI |  |  |  |  |
| No DSA | 1804 | Reference | | |
| <1k | 84 | 0.995 | 0.490-2.022 | 0.989 |
| 1-5k | 226 | 1.685 | 1.155-2.458 | **0.007** |
| 5-10k | 58 | 1.960 | 1.092-3.518 | **0.024** |
| >10k | 43 | 2.980 | 1.700-5.248 | **<0.001** |
| Rejections |  |  |  |  |
| ABMR | 174 | 3.483 | 2.582-4.699 | **<0.001** |
| TCMR | 379 | 2.223 | 1.698-2.911 | **<0.001** |
| HR: Hazard ratio, CI: confidence interval, MFI: Mean fluorescence intensity | | | |  |
