# Supplementary material for: The impact of pre-transplant donor specific antibodies on the outcome of kidney transplantation – Data from the Swiss transplant cohort study
Source: Front Immunol. 2022 Sep 21;13:1005790. doi: 10.3389/fimmu.2022.1005790 (PMC9532952; doi:10.3389/fimmu.2022.1005790)
Supplement: Supplementary file 4 [file Table_4.docx]

| **Supplementary Table 4. Summary of the investigated outcomes after kidney transplantation with and withour pre-transplant DSA, and the induction therapy applied on the DSA patients** | | | | |
| --- | --- | --- | --- | --- |
| **Parameter** | **All patients (n=2215)** | **DSA (n=411)** | **No DSA (n=1804)** | **p value (DSA vs. No DSA)** |
| ABMR | 174 (7.9%) | 106 (25.8%) | 68 (3.8%) | <0.0001 |
| TCMR | 379 (17.1) | 66 (16.1%) | 313 (17.4%) | 0.204 |
| Graft loss | 241 (10.9%) | 65 (15.8%) | 176 (9.8%) | <0.0001 |
| Mean eGFR (ml/min/1.73m2) | 52.4 | 50.4 | 52.9 | 0.737 |
| Mean eGFR slope (ml/min/1.73m2/year) | -0.50 | -0.96 | -0.39 | 0.005 |
| Mean total eGFR slope (ml/min/1.73m2/year) | -0.86 | -1.39 | -0.75 | 0.006 |
|  |  |  |  |  |
| **Pre-transplant DSA patients** | **Induction therapy** | | | |
| **Cumulative MFI** | **ATG/Thymo** | **Basiliximab** | **No induction** | **p value** |
| <1k | 51 (60.7%) | 32 (38.1%) | 1 (1.2%) | 0.3 |
| 1-2k | 75 (62.0%) | 46 (38.0%) | 0 | 0.252 |
| 2-5k | 78 (74.3%) | 27 (25.7%) | 0 | 0.543 |
| >5k | 71 (70.3%) | 30 (29.7%) | 0 | 0.244 |
