# Supplementary figures and images for: The impact of pre-transplant donor specific antibodies on the outcome of kidney transplantation – Data from the Swiss transplant cohort study
Source: Front Immunol. 2022 Sep 21;13:1005790. doi: 10.3389/fimmu.2022.1005790 (PMC9532952; doi:10.3389/fimmu.2022.1005790)

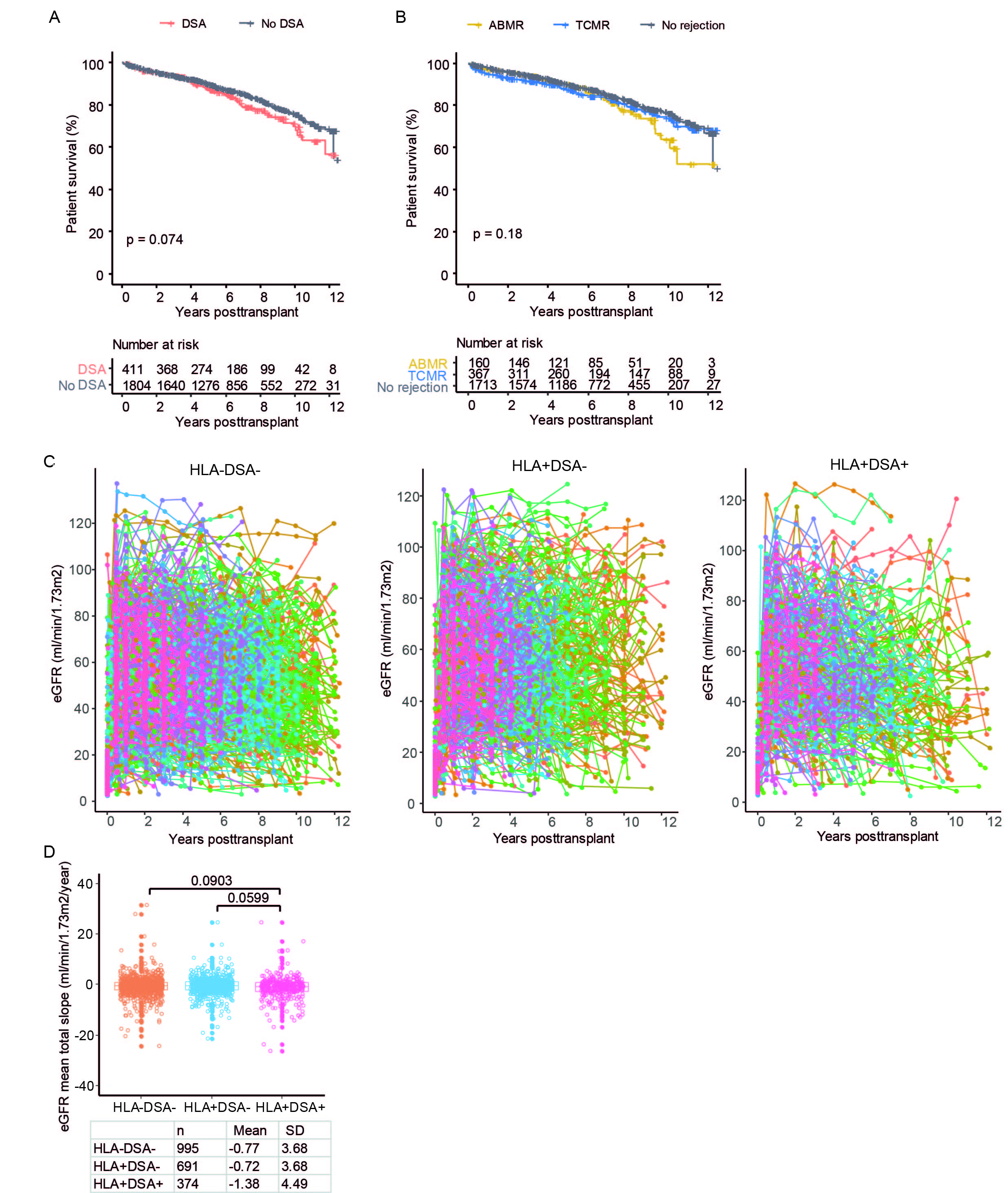

Supplement: Supplementary Figure 1 — The influence of HLA-DSA on kidney transplantation outcome. (A) Overall patient survival in patients with and without DSA. (B) Overall survival in patients that developed rejection (ABMR or TCMR) and in patients without rejection. The individual eGFR (C) trajectory plots in patients without anti-HLA antibodies (HLA-DSA-), with anti-HLA antibodies but without DSA (HLA+DSA-), and in patients with DSA (HLA+DSA+). (D) The collective mean total slope of eGFR in patients with HLA-DSA-, HLA+DSA- and HLA+DSA+, respectively. Log-rank test was used to test p value of the Kaplan-Meier survival curves for (A, B). One-way ANOVA (Kruskal-Wallis test) followed by Dunn’s post hoc test for (D). [file Image_1.jpeg]

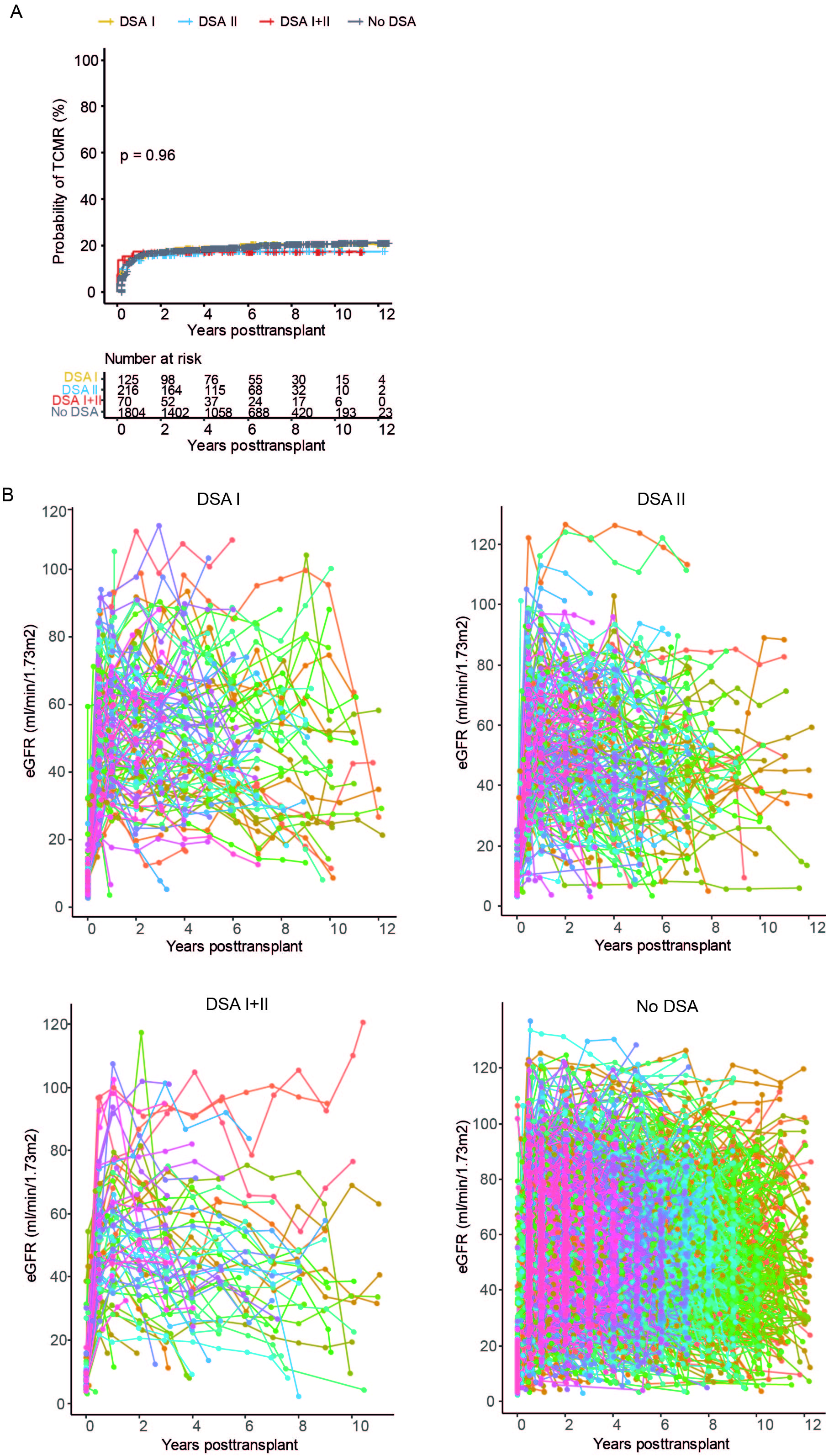

Supplement: Supplementary Figure 2 — The impact of DSA directed against Class I or Class II. Cumulative incidence of TCMR (A) and the individual eGFR trajectory (B) in in the patient groups with DSA directed against HLA Class I (DSA I), HLA Class II (DSA II), or a combination of Class I and Class II (DSA I+II) and in patients with no DSA. Log-rank test was used to test p value of the Kaplan-Meier survival curves for (A). [file Image_2.jpeg]

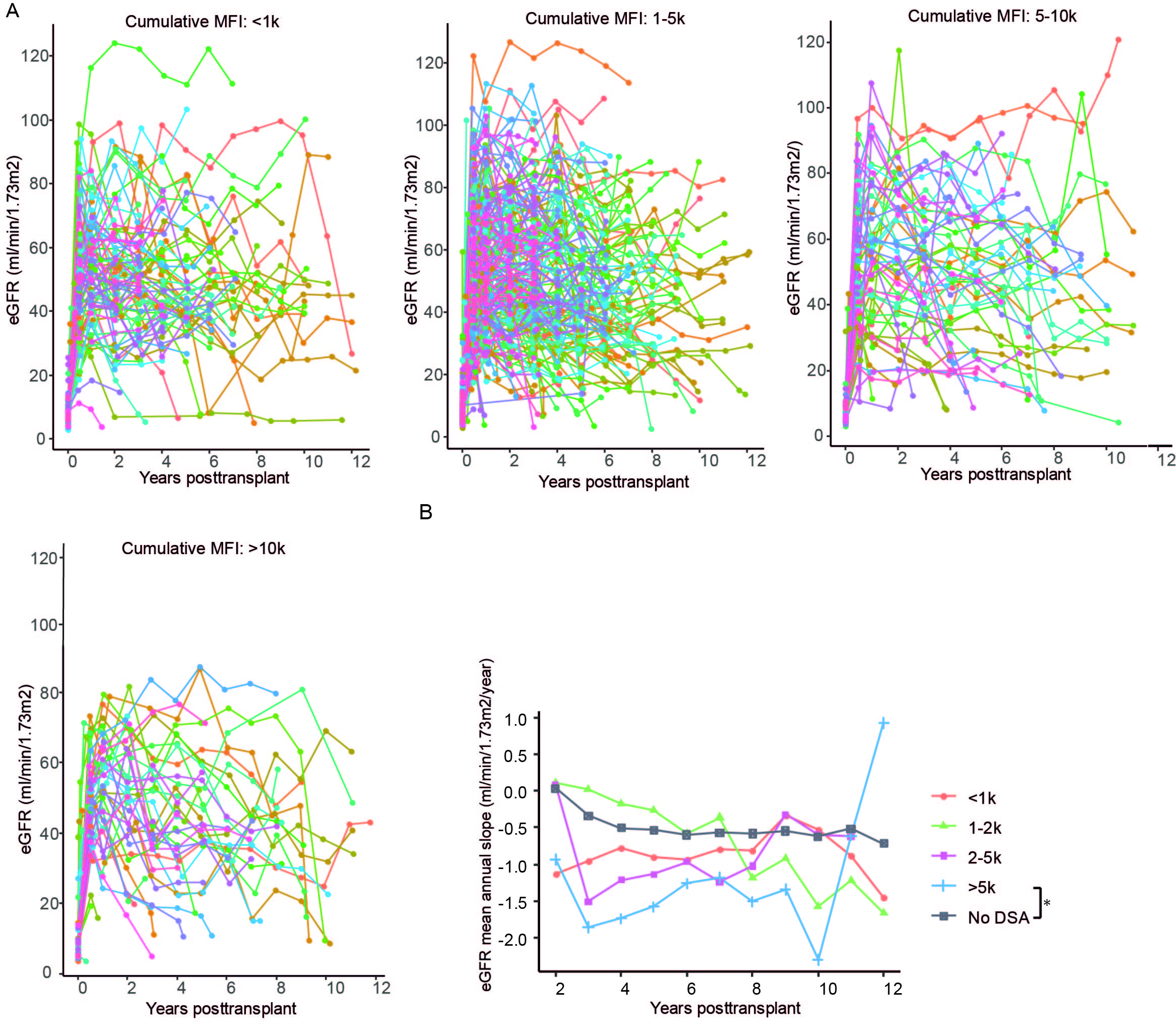

Supplement: Supplementary Figure 3 — The impact of MFI on the eGFR slope. (A) The individual eGFR trajectories in the patient groups with a cumulative DSA MFI of <1k, 1k-5k, 5k-10k, >10k, and no DSA. (B) The collective mean annual slope of eGFR in groups stratified on the single highest MFI of the detected DSA into <1k, 1k-2k, 2k-5k >5k, and no DSA groups. Two-way ANOVA analysis with Sidak’s multiple comparisons as a post hoc test were used for (B) to assess p values; *p<0.05, **p<0.01, [file Image_3.jpeg]

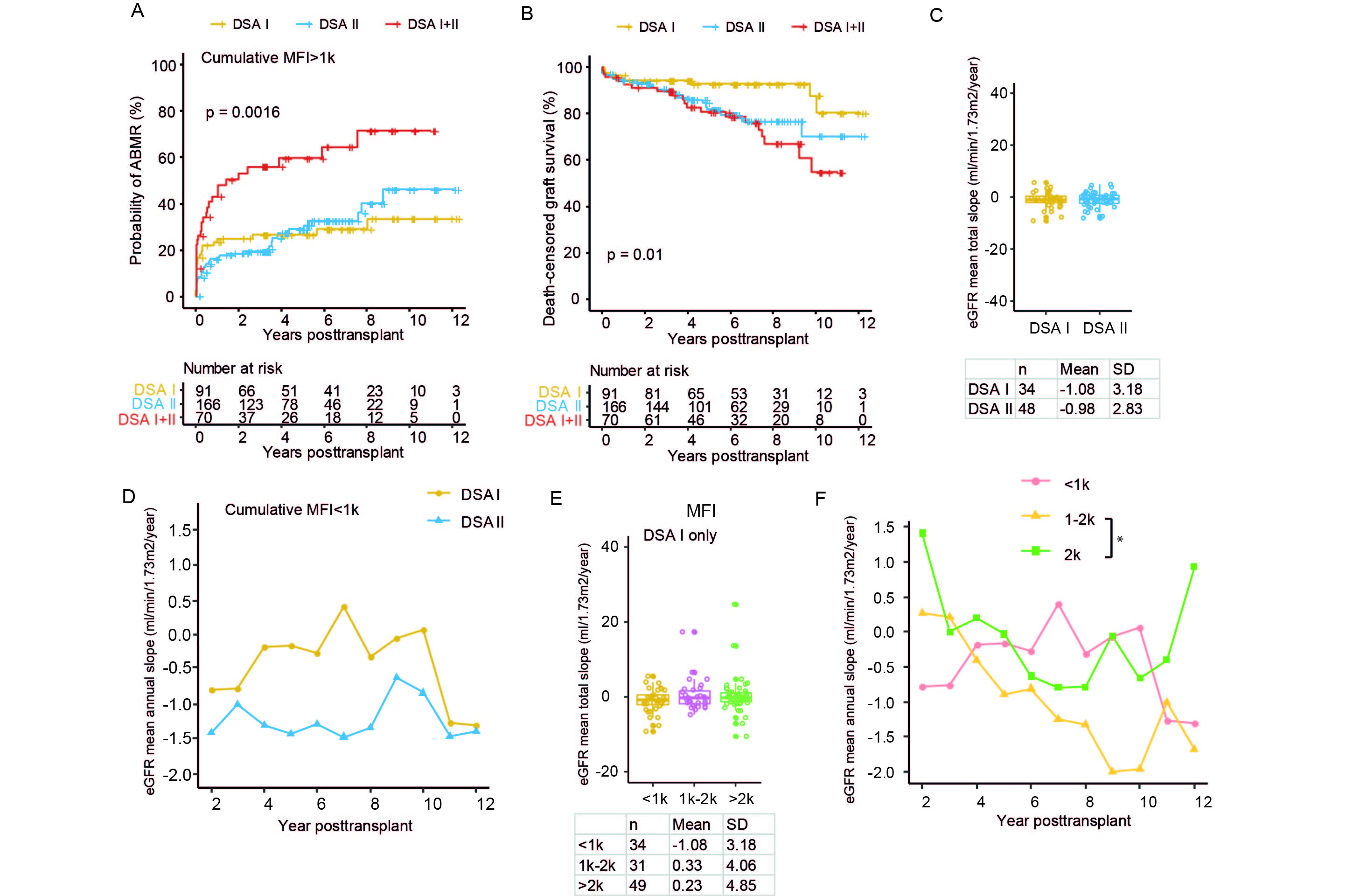

Supplement: Supplementary Figure 4 — Different DSA Classes differentially affect the outcome of kidney transplantation. Cumulative incidence of ABMR (A) and death-censored graft survival (B) in the DSA I, DSA II, DSA I+II groups with a cumulative MFI value >1k. The collective mean total slope of eGFR (C) and mean annual slope (D) in the DSA I and DSA II groups with cumulative MFI <1k. The collective mean total slope of eGFR (E) and mean annual slope (F) of patients with only DSA I in groups stratified on MFI into <1k, 1k-2k, and >2k. Log-rank test was used to test p value of the Kaplan-Meier survival curves for (A, B). One-way ANOVA followed by Dunn’s post hoc test for (C) and (E), two-way ANOVA analysis with Sidak’s multiple comparisons as a post hoc test were used for (D) and (F) to assess p values; *p<0.05. [file Image_4.jpeg]

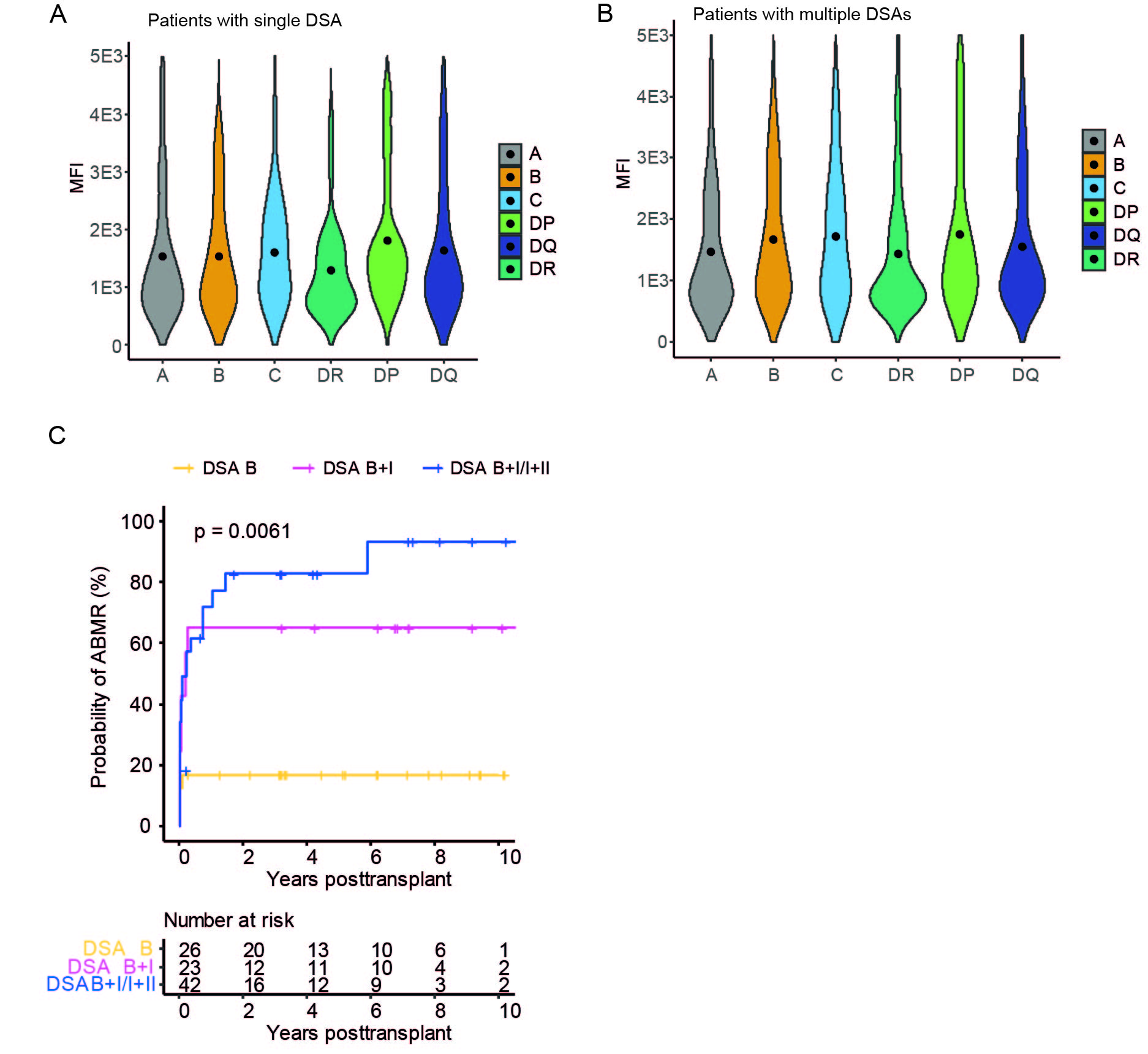

Supplement: Supplementary Figure 5 — DSA MFI overview and cumulative incidence of ABMR DSA combinations containing DSA against HLA-B. Violin plots of all detected DSA grouped into target HLA loci for patients with a single DSA (A) or with multiple DSA (B). (C) Cumulative incidence of ABMR in the presence of DSA directed against HLA-B only (DSA B only), or in patients with DSA directed at HLA-B + another Class I loci (DSA B + DSA I) and in patients with DSA directed at HLA-B + another Class II locus or a combination of additional Class I and Class II loci (DSA B + DSA II/I+II). One-way ANOVA (Kruskal-Wallis test) followed by Dunn’s post hoc test for (A, B) to assess the p value. Log-rank test was used to test p value of the Kaplan-Meier survival curves for (C). [file Image_5.jpeg]

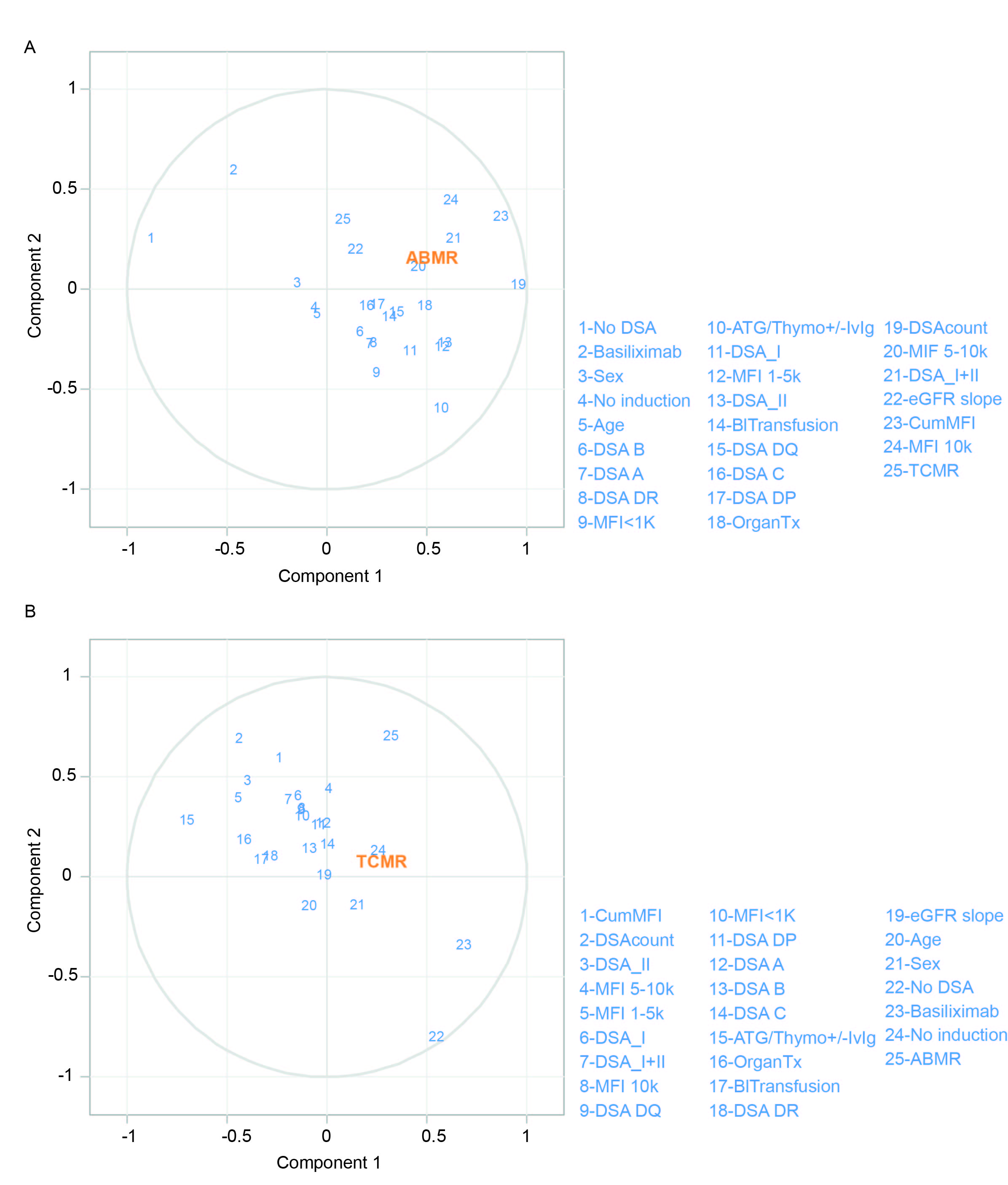

Supplement: Supplementary Figure 6 — Partial least squares (PLS) regression biplot for the first two components in ABMR and TCMR. Correlation shown between ABMR (A), TCMR (B) and the risk factors (in blue numbers). The first two axes which correspond to PLS components 1 and 2 are shown. The distance between the individual risk factors and the center indicates the strength of the correlation with each component and their alignments represent the correlation they contribute to the variation explained by each component. (OrganTx, Organ Transplantation; BlTransfusion, Blood Transfusion). [file Image_6.jpeg]
